# Identification of RNA binding motif proteins essential for cardiovascular development

**DOI:** 10.1186/1471-213X-11-62

**Published:** 2011-10-19

**Authors:** Samantha Maragh, Ronald A Miller, Seneca L Bessling, David M McGaughey, Marja W Wessels, Bianca de Graaf, Eric A Stone, Aida M Bertoli-Avella, John D Gearhart, Shannon Fisher, Andrew S McCallion

**Affiliations:** 1 Biochemical Science Division, National Institute of Standards and Technology, Gaithersburg, MD 20899, USA; 2 McKusick-Nathans Institute of Genetic Medicine, Johns Hopkins University School of Medicine, Baltimore, MD, 21205, USA; 3 CBG Department of Clinical Genetics, Erasmus Medical Centre, 3016 AH Rotterdam, The Netherlands; 4 Department of Genetics and Bioinformatics Research Center, North Carolina State University, Raleigh, NC, 27695-7566, USA; 5 Institute for Regenerative Medicine, University of Pennsylvania, Philadelphia, PA 19104, USA; 6 Department of Cell and Developmental Biology, University of Pennsylvania, Philadelphia, PA 19104, USA; 7 Department of Molecular and Comparative Pathobiology, Johns Hopkins University School of Medicine, Baltimore MD, 21205, USA

## Abstract

**Background:**

We recently identified *Rbm24 *as a novel gene expressed during mouse cardiac development. Due to its tightly restricted and persistent expression from formation of the cardiac crescent onwards and later in forming vasculature we posited it to be a key player in cardiogenesis with additional roles in vasculogenesis and angiogenesis.

**Results:**

To determine the role of this gene in cardiac development, we have identified its zebrafish orthologs (*rbm24*a and *rbm24b*), and functionally evaluated them during zebrafish embryogenesis. Consistent with our underlying hypothesis, reduction in expression of either ortholog through injection of morpholino antisense oligonucleotides results in cardiogenic defects including cardiac looping and reduced circulation, leading to increasing pericardial edema over time. Additionally, morphant embryos for either ortholog display incompletely overlapping defects in the forming vasculature of the dorsal aorta (DA), posterior caudal vein (PCV) and caudal vein (CV) which are the first blood vessels to form in the embryo. Vasculogenesis and early angiogenesis in the trunk were similarly compromised in *rbm24 *morphant embryos at 48 hours post fertilization (hpf). Subsequent vascular maintenance was impaired in both *rbm24 *morphants with substantial vessel degradation noted at 72 hpf.

**Conclusion:**

Taken collectively, our functional data support the hypothesis that *rbm24a and rbm24b *are key developmental cardiac genes with unequal roles in cardiovascular formation.

## Background

During vertebrate embryogenesis the heart is the first organ to develop and achieve functionality. Model organism studies have uncovered a number of genes such as Nkx2.5, which have important functions in early vertebrate myocardial development and differentiation [1,2]. Despite an increasing body of data illuminating the roles played by several key genes during cardiac development, there is still much to learn about what other factors may be critical. The general stages of cardiac development are invariable throughout vertebrate model organisms where heart development has been examined, with zebrafish progressing through these stages at a particularly rapid rate [3]. During zebrafish cardiogenesis the heart cone is the first structure to form between 19-20 hpf, followed by the formation of a short cardiac tube lacking discrete chambers by 24 hpf. Subsequently the cardiac tube lengthens and distinct ventricle and atrium chambers are discernable by 30 hpf with heart tube looping occurring around 36 hpf. A functional two chambered zebrafish heart is visible by 48 hpf [4].

In a recent transcriptional profiling study, we compared the signatures of mouse embryonic stem cells as they were differentiated towards cardiac cell fates in an effort to uncover novel critical cardiac genes. In this initial study we described the cardiac developmental expression of 31 identified candidate genes with previously unknown roles in cardiogenesis. Furthermore, nine of these transcripts were expressed in the forming cardiac crescent of the mouse embryo [5], consistent with roles in the earliest stages of heart development. Based on the early cardiac expression of these genes we predicted they likely play significant roles in heart development.

In this paper we report functional evaluation of one of these genes, *Rbm24*, through the identification, characterization and knockdown of its zebrafish (*Danio rerio*) orthologs. *Rbm24 *is a member of the RNA binding protein family, based upon the identification of a RNA recognition motif (RRM) in amino acid residues 12-84. RRMs are the most common and best characterized RNA binding modules with many functioning in most post-transcriptional processes [6]. The RRM is composed of four-stranded anti-parallel β-sheets with two helices packed against it such that the domain has the split αβ (βαββαβ) topology [7]. Often, RRMs function in concert to increase binding specificity possibly because the number of nucleotides recognized by a single RRM is generally too small to define a unique binding sequence [8]. RRMs are found in a variety of RNA binding proteins, including several hnRNP proteins, proteins implicated in regulation of alternative splicing, and protein components of snRNPs indicating a diverse role of these motifs in cellular development and function.

As we have shown previously, the mouse *Rbm24 *transcript is upregulated in the cardiac progenitor population and is expressed from the earliest stages of cardiac specification, in the cardiac crescent, and subsequently within the heart tube and looping heart [5]. Based upon these observations we hypothesized that *Rbm24 *plays a crucial role in cardiogenesis. In this study we investigated the putative role of Rbm24 in cardiac development. We have identified two *rbm24 *zebrafish orthologs (termed *rbm24a *and *rbm24b*), and demonstrate that their spatial expression is consistent with our observations in mice. We use *rbm24a *and *rbm24b *translation blocking morpholino antisense oligonucleotides (MO) in the early embryo and demonstrate that each zebrafish *rbm24 *ortholog has a key but unequal role to play in cardiac development. Additionally, we demonstrate a role for *rbm24a *and *rbm24b *in normal vascular development. Vascular development consists of vasculogenesis which is the *de novo *formation of the first vascular vessels and subsequently angiogenesis which is the formation of additional vasculature as extensions of existing vasculature. Zebrafish vasculogenesis results in the formation of the main trunk vessels with the DA first to form (24 - 26 hpf) followed by the CV and PCV (28 - 30 hpf) and several heart vessels generating the first single circulation loop. Angiogenesis can be observed in the trunk by 24 hpf as intersegmetal vessels (Se) begin to form as paired dorsally extending branches off the DA and subsequently off the PCV (~32 hpf). As the Se reach the dorsal line of the embryo the ends of the vessels form connections resulting in two dorsal longitudinal anastomotic vessels (DLAVs) running along the anterior-posterior (A-P) axis of the embryo at the dorsal line. By 48 hpf these trunk vessels are fully formed with circulation [9,10]. Our findings show the requirement for *rbm24a *and *rbm24b *in cardiac development and vasculogenesis with a more pronounced role for *rbm24a*, and an additional putative role for *rbm24a *in angiogenesis.

## Results

### Phylogenetic comparative analysis establishes two *rbm24 *orthologs in zebrafish

We recently reported the identification of *Rbm24 *as one of a number of novel transcripts discretely expressed in the earliest stages of cardiac development in the mouse embryo [5]. To facilitate determination of the biological requirement for this gene during vertebrate embryonic development, we first set out to identify the orthologous gene/s in zebrafish. In the transition between assembly Zv8 and Zv9 of the zebrafish genome, first one and then a second putative *rbm24 *ortholog was identified. The current assembly (Zv9) identifies a protein coding gene as *rbm24a *residing on chromosome 19 and also a novel annotation of a putative *rbm24b *protein coding gene residing on chromosome 16. To confirm the validity of these most recent changes to the genome annotation we compared these findings to our own comparative genomic analyses.

The first annotated *rbm24a *gene encodes a protein that displays strong similarity to the mouse Rbm24 (E-value = 3e-90; 188/237 (79%) amino acids). Using the mouse Rbm24 as a blastp query to search the NCBI RefSeq database of *D. rerio *proteins we detected strong similarity (E-value = 5e-61; 103/116 (88%)) to a hypothetical protein LOC562236 encoded by a gene (*zgc:136803*) on chromosome 16 (Zv8). This protein is now termed *rbm24b *in Zv9. The identification of these genes as *rbm24 *orthologs is further supported by the identification of a pair of annotated *rbm24 *paralogs in both the medaka (*Oryzias latipes*; chr. 11, 16) and pufferfish (*Tetraodon nigroviridis; *chr. 21 and 8) genomes. Zebrafish chromosome 19, where *rbm24a *resides, shares a common evolutionary origin with *O. latipes *chromosome 11 and *T. nigroviridis *chromosome 21 [11]. Similarly, chromosomes 16 and 8 in *O. latipes *and *T. nigroviridis*, respectively, map to the *rbm24b *region of chromosome 16 in *D. rerio*.

### *rbm24a *and *rbm24b *display cardiovascular expression during embryogenesis

**Figure 1:**
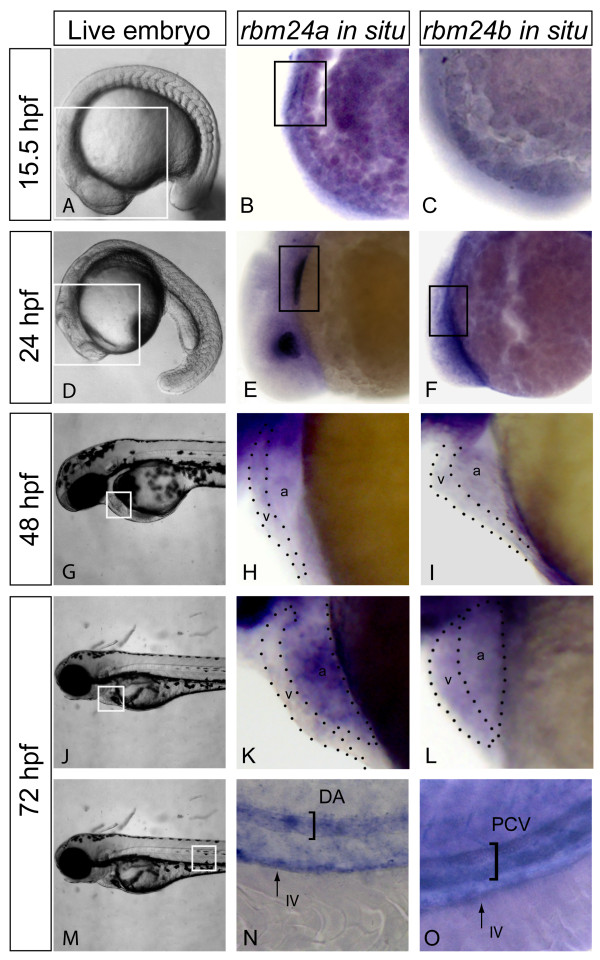
***rbm24a *and *rbm24b *display cardiovascular expression during embryogenesis**. Expression of *rbm24 *transcripts was evaluated in uninjected embryos fixed at 15.5, 24, 48 and 72 hpf. Live embryos 15.5 hpf with area of interest for rbm24 *in situ *boxed in white (A). Lateral heart views of *rbm24a *and *rbm24b in situs *on uninjected 15.5 hpf embryos showed expression linearly organized myocardial precursor cells for *rbm24a *(black box, B) but not *rbm24b *(C). Live embryos 24 hpf with area of interest for rbm24 *in situ *boxed in white (D). Lateral heart views of *rbm24a *and *rbm24b in situs *showed expression in the developing heart tube at 24 hpf (black box, E, F) with lens expression for *rbm24a *alone. 48 hpf live embryo showing the heart boxed in white (G). Lateral zoom of the heart showed *rbm24a *and *rbm24b *were expressed in the ventricle (v) and atrium (a) of the looped heart at 48 hpf (H, I). Live 72 hpf embryos with the heart boxed in white (J). Expression of both *rbm24 *transcripts was detected in the heart at 72 hpf (K, L). Live image of a 72 hpf embryo with the area of interest for vascular expression boxed in white (M). Expression of both *rbm24 *transcripts was detected in the trunk vasculature with differing expression patterns. *rbm24a *shows arterial expression in the DA (N) while *rbm24b *shows venous expression in the PCV (O) with both being expressed in the IV. DA, dorsal aorta; PCV, posterior caudal vein; IV, intestinal vasculature.

The spatial and temporal expression of both *rbm24 *orthologs was determined using RNA *in situ *hybridization (ISH) (Figure 1). Both *rbm24 *orthologs show cardiac expression early in development. At 15.5 hpf we detected *rbm24a *but not *rbm24b *expression in myocardial precursor cells (Figure 1B and 1C). By 24 hpf both *rbm24a *and *rbm24b *expression was detected in the forming heart (Figure 1E and 1F). Expression of *rbm24a *was also detected in the lens of the eye. By 48 hpf, and continuing through 72 hpf, *rbm24a *and *rbm24b *exhibit broad expression throughout the heart, although expression of *rbm24a *was higher in the atrium than the ventricle. By contrast expression of *rbm24b *appeared uniformly expressed in both heart chambers (Figure 1I and 1L). These data are consistent with cardiac expression of the mouse *Rbm24 *ortholog, and suggest incompletely overlapping requirements for *rbm24a *and *rbm24b *during zebrafish cardiac development.

By 72 hpf both *rbm24a *and *rbm24b *show incompletely overlapping vascular expression in addition to cardiac expression. *rbm24a *is expressed in the DA and intestinal vasculature (IV) while *rbm24b *is expressed in the PCV as well as the IV (Figure 1N and 1O). The early expression of both *rbm24a *and *rbm24b *in both the heart and vasculature suggests potentially important roles in cardiogenesis and vasculogenesis.

### *rbm24a *and *rbm24b *are required for normal cardiac development

**Figure 2:**
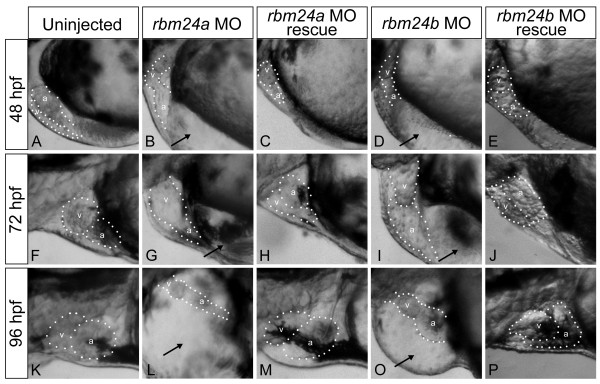
***rbm24a *and *rbm24b *are required for normal cardiac development**. Translation blocking morpholinos complementary to *rbm24a *(5 ng) *or rbm24b *(8 ng) were injected into 1-2 cell stage zebrafish embryos and the resulting phenotypes were evaluated compared to uninjected controls at 48 hpf (A, B, D), 72 hpf (F,G,I), and 96 hpf (K, L, O). Lateral heart views are shown with a dotted outline around the heart chambers. Both morphant embryo conditions exhibited cardiac looping defects and edema at all stages. Heart chambers are shown with a dotted outline with chambers denoted: v, ventricle; a, atrium; black arrows, cardiac edema. Phenotype rescue was achieved for each *rbm24 *via co-injection of each respective full length capped poly-A RNA transcript (*rbm24a *800 pg, *rbm24b *50 pg) along with the respective complementary translation blocking morpholino into 1-2 cell stage embryos where 800 pg *rbm24a *(C, H, M) or 50 pg *rbm24b *(E, J, P) achieved rescue. Rescued embryos posses looped hearts absent of edema.

To determine the functional role of the *rbm24 *orthologs in cardiac and vascular development, translation blocking morpholino antisense oligonucleotides (MO) were designed against the 5' UTR of each transcript. The effects of gene knockdown was assessed for each ortholog individually post microinjection of either MO into zebrafish embryos at the 1-2 cell stage. Titration experiments were conducted to determine MO quantities sufficient to induce a consistent phenotype in a majority of embryos injected (Additional File 1). Injection of 5 ng of *rbm24a *MO or 8 ng *rbm24b *MO resulted in a robust cardiac phenotype for a significant number of injected embryos at 48 hpf (64/67 and 59/62 respectively) (Table 1), compared to uninjected embryos (Figure 2A, B and 2D). During cardiogenesis the heart tubes of all affected morphant embryos failed to correctly loop, remaining linear but with distinct ventricular and atrial chambers (Figure 2). Affected embryos also exhibited reduced blood circulation, which led to cardiac edema in all cases by 48 hpf and continually worsened as development progressed (Additional File 2, 3, 4, 5, 6, 7, 8, 9, 10). A subset of *rbm24a *MO treated embryos (6/64) displayed an even more severe phenotype, lacking a distinct heart tube and exhibiting a beating focus of periodically contracting cells. This poorly organized cardiac structure was located ventral to the embryo between the embryo and the yolk suggesting a defect in the migration and organization of the cardiac fated cells. Similarly reduction of *rbm24a *or *rbm24b *expression using splice blocking morpholinos, designed against the second intron/exon boundary, also resulted in cardiac looping defects and cardiac edema in a majority of embryos (92.7% and 54.4% respectively) (Additional File 11). Knockdown efficiency was evaluated by RT-PCR for embryos displaying cardiac looping defects and cardiac edema after injection with splice blocking morpholino. Both rbm24a and rbm24b transcript levels were significantly reduced to 42.80% +/- 3.35, P < 6.5 × 10^-4 ^and 40.27% +/- 3.19, P < 4.4 × 10^-5 ^of transcript levels in respective uninjected controls (Additional File 12). We used translation blocking MO in all subsequent MO-based experiments due to their greater efficacy.

**Table 1:** Number of embryos with cardiac defects in *rbm24 *morphant and rescue conditions

Morpholino	Dosage	Embryos Studied	Looping Defects	Cardiac Edema	No Cardiac Organization
*rbm24a *MO	5 ng	67	58 (86.6%)	64 (95.5%)	6 (9%)
*rbm24a *rescue	+ 800 pg RNA	45	9 (20%)	9 (20%)	0
*rbm24b *MO	8 ng	62	59 (95.2%)	59 (95.2%)	0
*rbm24b *rescue	+ 50 pg RNA	47	12 (25.5%)	12 (25.5%)	0

To confirm the phenotype observed in the *rbm24 *morphants was not due to non-specific morpholino toxicity, embryos were co-injected with *p53 *morpholino and *rbm24a *MO or *rbm24b *MO (Methods). The cardiac phenotypes remained unchanged in the presence of the *p53 *morpholino (Additional File 13). We then performed phenotype rescue for both *rbm24 *morphants to determine the sequence specificity of the observed morphant phenotypes. We generated full length capped poly-A RNA transcripts for each ortholog and co-injected each along with the respective *rbm24 *MO, directed against the 5'UTR not present in the *in vitro *transcribed RNA. Rescue for *rbm24a *MO was achieved with 800 pg of RNA (36/45) while 50 pg of RNA was sufficient for *rbm24b *MO rescue (35/47). For both orthologs rescue resulted in properly looped hearts, normal systemic circulation and an absence of cardiac edema and by 72 hpf and continuing to 96 hpf (Figure 2F, H, J, K, M and 2P). Taken collectively, these data suggest the phenotype seen with each *rbm24 *MO is a specific result of reduction in gene expression and support the inference from ISH expression data that both *rbm24 *orthologs are required for normal cardiac development.

### Depletion of *rbm24a *and *rbm24b *compromise cardiac myocardium development

**Figure 3:**
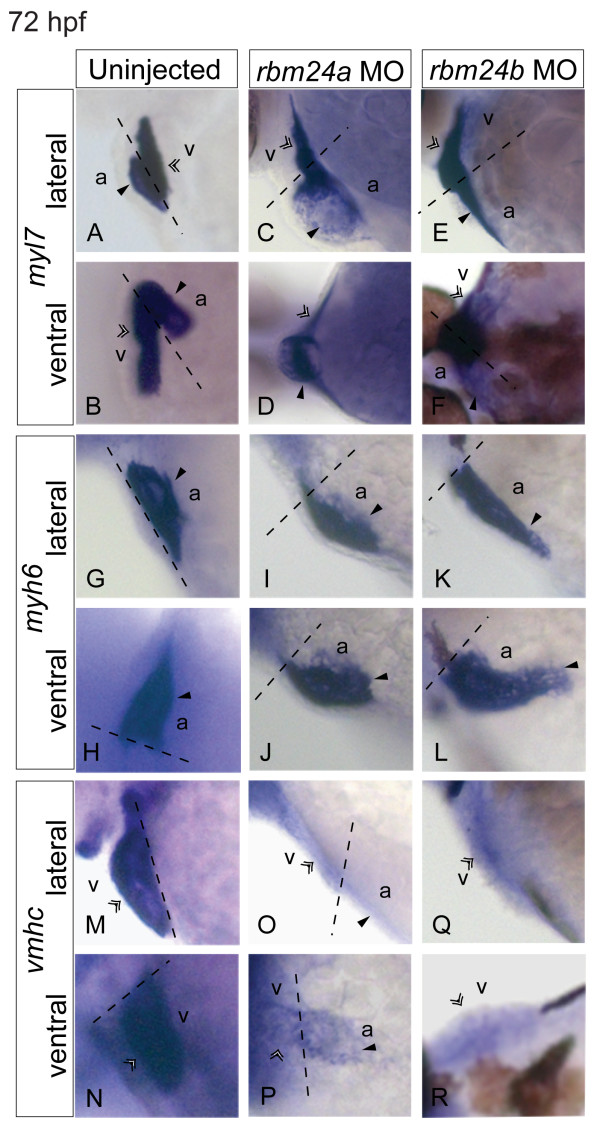
**Depletion of *rbm24a *and *rbm24b *compromise cardiac myocardial development**. The expression patterns of cardiac markers were analyzed in *rbm24 *morphants and uninjected controls at 72 hpf. Ventral and lateral images of the heart are shown with a dotted line marking the boundary between heart chambers. By *myl7 *expression of the entire heart, uninjected controls possess looped hearts with defined ventricle and atrium chambers (A, B) in contrast to unlooped hearts for both *rbm24 *morphants. *rbm24a *morphants displayed linear presumptive ventricle and an incompletely formed presumptive atrium (C, D). *rbm24b *morphants displayed a linear presumptive atria and ventricle (E, F). *myh6 *marking the atrium displayed a looped atrium in controls (G, H) with linear atrium in *rbm24*a (I, J), and *rbm24b *morphants (K, L). *vmhc *expression demarks looped ventricles in controls (M, N). *rbm24a *morphants displayed little expression bound to an unlooped heart tube where expression extends to the presumptive atrium (O, P). Almost no *vmhc *expression is detectable in *rbm24b *morphants (Q, R). v, ventricle region; double arrow, ventricular expression a, atrium region; solid black arrow, atrial expression.

To better evaluate the impact to the heart of reducing *rbm24a *or *rbm24b *we assayed the expression of several key myocardial transcripts by ISH, including myosin light polypeptide 7, *myl7 *(heart), myosin heavy polypeptide 6, *myh6 *(atrium) and ventricular myosin heavy chain, *vmhc *(ventricle). Expression of each marker was evaluated in control (uninjected), *rbm24a *MO and *rbm24b *MO injected embryos fixed at 72 hpf at which point the zebrafish heart is normally, looped, fully developed and functioning, allowing us to comment in greater detail on the structural deficits contributing to the gross morphological morphant phenotypes. When examining the entire heart, both *rbm24a *and *rbm24b *morphant embryos exhibit strong *myl7 *expression levels comparable to that of controls albeit within an unlooped heart tube. The hearts of controls by contrast are appropriately looped (Figure 3A - F). Additionally in *rbm24a *morphants defects of the presumptive atrium of the forming heart tube appear more severe than in the ventricular portion (Figure 3C and 3D). By contrast organization of the developing ventricle and atrium in *rbm24b *MO embryos appears equally compromised (Figure 3E and 3F). Expression of the atrial marker *myh6 *for both *rbm24 *morphants appears qualitatively similar to levels in uninjected controls. Presumptive atria of *rbm24a *morphant heart tubes appear linear and truncated, while to *rbm24b *morphants also have linear longer heart tubes, both contrasting with the looped atrium observed in uninjected controls (Figure 3G - L). These data are consistent with the observations that *rbm24a *expression was higher in the atrium than the ventricle by 72 hpf, contrasting with the more uniform expression observed for *rbm24b *across both heart chambers (Figure 1).

Both *rbm24 *morphant conditions exhibited a near abrogation of the levels of the ventricular marker *vmhc *compared to strong expression in uninjected controls (Figure 3M - 3R). In *rbm24a *MO morphants expression of *vmhc *was faint but with expression clearly bounded to the heart tube with a clear ventricular portion; however, the expression uncharacteristically extended beyond the ventricular portion into the presumptive atrium of the heart tube (Figure 3O and 3P). For *rbm24b *morphants *vmhc *expression was very diffuse and failed to clearly mark a ventricular boundary. These findings for *vmhc *expression were unexpected; both in the reduction of total expression seen for both *rbm24 *morphants and the apparent stronger severity of the ventricular phenotype compared to the atrial phenotype for both *rbm24 *morphants. Taken together the expression of these markers highlight the differential spatial impact of each *rbm24 *ortholog in heart structure development of the myocardium, and is consistent with our previous gross morphological observations. These data indicate an incompletely overlapping role for *rbm24a *and *rbm24b *in heart tube formation and subsequent heart looping.

### Edema in morphant embryos may have multiple origins

**Figure 4:**
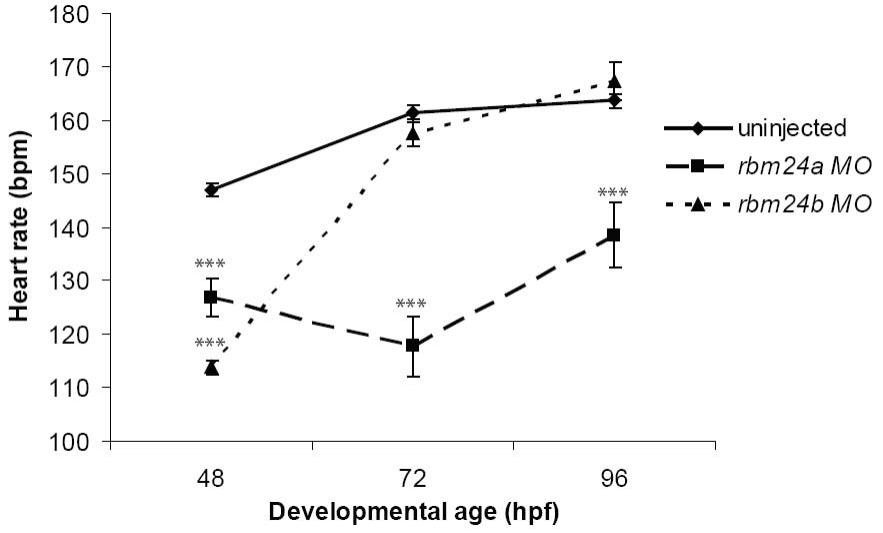
**Slow heart rate may contribute to cardiac edema in *rbm24 *morphants**. Heart rates were determined for uninjected control, *rbm24a *and *rbm24b *morphant embryos at 48, 72 & 96 hpf and significance was ascribed from a t-test statistic. 50 embryos were examined for each condition at each time point. At all time-points the heart rate of *rbm24a *morphants was significantly lower than controls where *P *< 2 × 10^-5^. At 48 hpf *rbm24b *morphants had significantly lower heart rates than controls (*P *= 1 × 10^-32^). The heart rates for *rbm24b *morphants then rose to rates not different from controls by 72 hpf and 96 hpf (*P *> 0.12) while maintaining the features of the morphant phenotype. Error bars here are standard error. *** *P *< 0.001.

The observed cardiac edema may result from reduced circulation as a consequence of defects in heart structure, heart rate, aberrant vasculature or combinations of these factors. Given the observed structural defects, we additionally assayed heart rate as a potential contributor to cardiac edema in the *rbm24a *MO and *rbm24b *MO injected embryos [12]. Heart rate counts were determined for uninjected, *rbm24a *MO and *rbm24b *MO injected embryos (n = 50) at 48, 72 and 96 hpf. At 48 hpf both *rbm24a *and *rbm24b *morphants displayed significantly lower heart rate compared to uninjected controls (P < 0.0002) (Figure 4). Heart rates in *rbm24a *MO injected embryos remain significantly lower than uninjected controls at 72 hpf and 96 hpf. By contrast the heart rate of *rbm24b *MO injected embryos recovered such that it did not differ significantly from uninjected controls at 72 hpf and 96 hpf (P > 0.12) despite their continued structural anomalies. These data demonstrate cardiac edema is not ameliorated in *rbm24b *morphants despite heart rates increasing to normal levels, suggesting the role of other factors must be considered as contributing to compromised circulation. Although defects in structure and rates of contraction may contribute to compromised circulatory function, we posit that vascular disruption likely also plays a role in the resulting edema.

### *rbm24a *and *rbm24b *are required for normal vasculogenesis with a potential role in early angiogenesis

**Figure 5:**
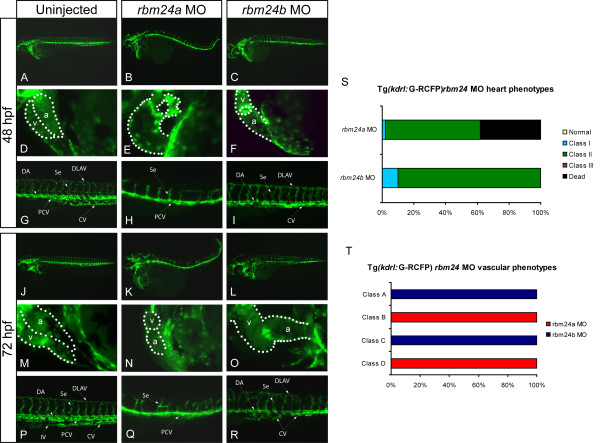
**Reduction of *rbm24a *or *rbm24b *results in aberrant vasculature and a lack of vascular maintenance**. TG(*kdrl*:G-RCFP) zebrafish line was used to asses the impact of *rbm24 *depletion on vasculature at 48 hpf and 72 hpf. Lateral views are shown for whole embryo, heart and trunk with a dotted outline indicating heart chamber boundaries. Uninjected controls at all time-points display looped hearts and properly developed vasculature. (A, D, G, J, M, P). 48 hpf *rbm24a *morphants possess unlooped hearts but display only the PCV and stunted disorganized Se (B, E, H). 48 hpf *rbm24b *morphants possess unlooped hearts, and only the DA and CV are visible with disorganized Se (C, F, I). By 72 hpf there was increased vascular disorganization and degeneration and a lack of heart looping and in both *rbm24 *morphants (K, N, L, O, Q, R). Cardiac morphant phenotypes are displayed quantitatively as percentages; Normal, looped beating heart with no cardiac edema; Class I, looped beating heart with cardiac edema. Class II, unlooped beating heart tube with cardiac edema, Class III, beating heart cell mass with cardiac edema; Dead, extreme cell death and degradation of embryo (S). Morphant vascular phenotype classes; Class A, disorganized trunk vasculature with formed DLAV; Class B, disorganized trunk vasculature with truncated Se and no DLAV; Class C, no discernable PCV; Class D, no discernable DA or CV (T). v, ventricle; a, atrium; DA, dorsal aorta; CV, caudal vein; PCV, posterior caudal vein; Se, intersegmental vessels, DLAV, dorsal longitudinal anastomotic vessels; IV, intestinal vasculature.

To determine the functional role of *rbm24a *and *rbm24b *in vascular development we also undertook MO-based analyses of each ortholog in the TG(*kdrl*:G-RCFP) transgenic zebrafish reporter line which fluorescently marks all endothelial cells highlighting the forming cardiovascular structures [13]. Embryos were examined for vascular expression, after injection of *rbm24a *MO or *rbm24b *MO as described above, after heart formation and completion of vasculogenesis at 48 and 72 hpf (Figure 5). As per our earlier experiments, morphant embryos for both genes exhibit endocardial cardiac edema and a lack of heart looping displaying little to no circulation with 30/31 and 45/50 embryos displaying cardiac defects for *rbm24a *and *rbm24b *morphants respectively (Figure 5D - F and 5M - O). Additionally, all *rbm24a *and *rbm24b *morphant embryos display a reduction in total trunk vasculature and vascular organization in 100% of the embryos (Figure 5G - I and 5P - R); however vasculogenic and angiogenic vessels are disrupted to differing degrees. At 48 hpf *rbm24a *morphants posses a clearly present PCV appearing thicker than normal with no detectable DA or CV (Figure 5H). Angiogenesis is severely disrupted in these embryos with unpaired stunted dysmorphic Se also appearing thicker extending only to the midline of the embryo and thus a complete lack of DLAVs. By contrast the *rbm24b *morphant at 48 hpf shows little to no disruption of the DA, a visible but malformed CV and no detectable PCV (Figure 5 I). Unlike *rbm24a *morphants, *rbm24b *morphants have less disruption of angiogenesis. Se in *rbm24b *morphants display pairing at the posterior end of the embryo and extend dorsally the length of the embryo forming one DLAV; however, all these vessels are structurally dysmorphic. Both *rbm24a *and *rbm24b *vascular morphant phenotypes become progressively more severe between 48 hpf and 72 hpf. Vessels are less well organized and the number of vessels present is reduced, with no evidence of IV (Figure 5J - L and 5P - R). Of the morphant embryos displaying cardiac defects *rbm24a *and *rbm24b *morphants segregated into different classes of vascular defects, with 31/31 *rbm24a *morphants analyzed possessing disorganized trunk vasculature where Se do not extend the entire dorsal length of the embryo and the DA and CV are undetectable; and 50/50 *rbm24b *morphants had disorganized trunk vasculature where the Se formed DLAV and yet the PV was undetectable (Figure 5S and 5T). These data correlate with respective primary vascular expression locations of *rbm24a *of DA and *rbm24b *of PCV and both displaying expression in the IV (Figure 1G and 1H). When expression of *rbm24a *or *rbm24b *is compromised formation of the vessels required for the initial circulation loop is similarly compromised, likely contributing significantly to the observed cardiac edema in morphant embryos. These findings indicate the endocardium is also compromised as a result of *rbm24 *knockdown and also suggests that both *rbm24a *and *rbm24b *are independently necessary for normal early vascular development and support a putative role for *rbm24a *in angiogenesis.

### *rbm24a *and *rbm24b *exhibit incompletely overlapping functions in zebrafish development

**Figure 6:**
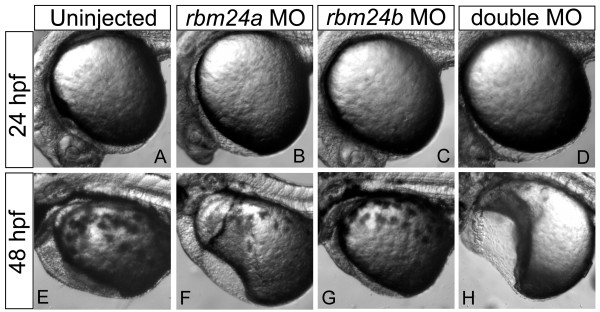
**Double *rbm24a *and *rbm24b *morphants display more severe cardiac defects**. Morpholinos directed to each *rbm24 *ortholog were injected individually and in tandem into zebrafish embryos and assessed at 24 hpf and 48 hpf compared to uninjected embryo hearts (A and E). Individual morpholino injections were titrated to a low dose for both *rbm24a*, 2.5 ng (B and F) and *rbm24b*, 4ng (C and G). Embryos receiving low doses of both *rbm24a *and *rbm24b *morpholinos resulted in more severe phenotype than either low dose MO alone, exhibiting severe cardiac defects and increased edema (D and H).

To determine whether *rbm24a *and *rbm24b *interact genetically in the developing zebrafish embryo, we injected low doses of both *rbm24a *and *rbm24b *MOs into the same embryo. Although each MO dosage amount was too low (2.5 ng and 4 ng respectively) to elicit a strong consistent phenotype alone as evidenced by titration experiments (Additional File 1), a much more severe phenotype resulted in the morphants receiving both MOs in concert (Figure 6). The double morphants displayed greater cardiac edema at 24 hpf than either morphant alone (Figure 6A - D). By 48 hpf, the double morphants also displayed very little cardiac organization, with most (73/93) failing to form any heart tube structure. No double morphants displayed any circulation (Figure 6E- H). This severe and distinctive phenotype exceeds that observed for either MO when assayed independently, even at the higher concentrations that yield consistent cardiac defects (5 ng of *rbm24a *MO or 8 ng *rbm24b *MO). These data support the possibility that roles for *rbm24 *orthologs may overlap during cardiovascular development.

## Discussion

*Rbm24 *is a novel cardiac gene candidate identified through transcript profiling of differentiating mESCs [5]. It was initially selected for functional analysis due to its early cardiac expression during mouse development, first detected by *in situ *hybridization in the cardiac crescent of mice, and subsequently in the heart tube and looping heart.

We integrated syntenic and sequence-based comparative genomic analyses to identify two *rbm24 *orthologs in zebrafish, establishing that their expression was concordant with our earlier observations in mice. In the zebrafish embryo, a reduction of either *rbm24 *ortholog expression by injection of morpholinos results in cardiac looping defects and reduced circulation. These embryos later exhibit cardiac edema and distention as early as 48 hpf, but this is likely a secondary effect of reduced *rbm24 *expression, due instead to reduced circulation [14]. Our data suggests that reduced circulation is likely due to a reduction in critical vasculature and not simply reduced heart rate. Additionally, reduction of either *rbm24a *or *rbm24b *independently yielded vascular defects of both myocardium and endocardium. We exclude general toxicity or off target effects as the cause of the observed morphant phenotype by confirming they remain without amelioration in the presence of *p53 *MO. We also confirmed the morphant phenotypes are sequence-specific by successfully achieving RNA-based phenotype rescue.

Investigation of molecular markers for myocardial development further illuminated the impact of reducing *rbm24 *expression. Both *rbm24 *orthologs are detected in the developing heart by *in situ *hybridization by 24 hpf and sustain expression at least through 72 hpf. In all cases a reduction of *rbm24a *or *rbm24b *was sufficient to prevent heart looping noted at 48 hpf and beyond, where normal heart looping occurs at approximately 36 hpf. Expression of *rbm24a *is unequal between the ventricle and atrium with stronger expression in the atrium while *rbm24b *shows equivalent expression in both chambers. This skewed expression of *rbm24a *at higher levels in the atrium is illustrated by truncated and abnormal atrium formation of the developing heart in *rbm24a *MO injected embryos demonstrating its requirement for cardiogenesis, including atrial specification, at this early stage. A reduction in either *rbm24 *ortholog severely disrupts the expression level and localization of ventricular marker *vmhc *yielding little to no expression visible in either *rbm24 *morphant condition. Reduced ventricular myocardial count disproportionate to the atrium has also been reported with deficient *nkx *and hedgehog signaling [15,16]. Although reduction of each *rbm24 *results in incompletely overlapping atrial and ventricular phenotypes, reduction of either is sufficient to compromise the looping of the heart tube. The looping of the heart tube is a result of both genetic and biophysical mechanisms, and perturbation of the mechanical force generated by the contraction of the heart chambers can affect proper heart formation [17]. Zebrafish *wea *mutants (*myh6*), display distortions in cardiac looping, but still maintain a functional circulatory system due to the adaptation of the ventricle [18]. This suggests that reduction in *rbm24a *expression impacts both the ventricle and atrium, likely in the organization or structure of the muscle filaments, in addition to cardiomyocyte count, in turn affecting the ability of the heart to circulate blood.

Although both *rbm24a *MO and *rbm24b *MO injected embryos display an additional phenotype of aberrant vascular morphology, with defects in vasculogenesis and angiogenesis the defect in *rbm24a *morphants appears more severe. The observed phenotypes, however, are also consistent with the exhibited localization of each *rbm24 *ortholog, wherein *rbm24a *is expressed in the DA and *rbm24b *is expressed in the PCV by 72 hpf. The expression difference likely reflects the acquisition of independent roles since duplication of the ancestral gene [19,20], resulting in incompletely overlapping functions for these orthologs in vasculogenesis. Angiogenesis is impaired when expression of either *rbm24 *ortholog is reduced. The integrity of these first vessesls of angiogenesis (Se and two DLAVs) deteriorates over time. In *rbm24a *morphants Se are more severely affected not reaching the dorsal line to form DLAVs at 48 hpf and continue to deteriorate by 72 hpf. This may result form the lack of any discernable DA. *rbm24b *morphants still possessed a visible DA but no PCV, Se and one DLAV were present and only displayed mild morphology alterations at 48 hpf with subsequent severe deterioration by 72 hpf. In the case of all *rbm24 *morphants there is an inability to maintain the new vessels formed during angiogenesis and a wasting of any vessels resulting from vasculogenesis. It is unclear the molecular basis of this blood vessel deterioration. Normal *rbm24 *expression may be playing a direct role in the vascular maintenance or the angiogenesis pathway. Also to be considered is that a lack of sufficient concentrations of growth factors and mitogens due to insufficient circulation may be preventing maintenance of initiated vasculature, since it has been shown Se sprouting and extension involves several rounds of cell division [21] and *vegfA *is required for vascular maintenance [22].

The incompletely overlapping role of *rbm24a *and *rbm24b *in the developing heart and vasculature is underscored by the extreme phenotype observed in the double morpholino injections. *rbm24a *and *rbm24b *appear to act in parallel in cardiac development, converging on a common phenotype for which they are both overtly required. By contrast in vasculogenesis, *rbm24a *and *rbm24b *appear to function in parallel but differentially in artery and vein formation, resulting in different vascular phenotypes which are individually sufficient to disrupt formation of the initial embryonic circulation loop. Further analyses will be required to determine the mechanistic role played by *rbm24a/b *and whether their roles involve one or all of the above-discussed mechanisms.

The critical role for *rbm24 *in cardiogenesis raises the question whether mutations at the orthologous human locus may contribute to cardiac disorders. Interestingly, Wessels *et al. *recently identified a novel cardiac syndrome with noncompaction cardiomyopathy, bradycardia, pulmonary stenosis, atrial septal defects, and heterotaxy with genetic linkage to human chromosome 6p [23]. Genome-wide linkage analysis localized the implicated interval to chromosome 6p24.3-21.2, a region encompassing the human *RBM24 *ortholog (6p22.3), making it an ideal candidate for variation underlying this phenotype. However, sequencing of all *RBM24 *coding exons, intron/exon boundaries and the upstream promoter region in two affecteds, revealed only known polymorphisms (rs10456798 and rs35860841) that were also observed in a heterozygous state in an unaffected family member. While we can exclude a role for *RBM24 *coding mutations, additional sequencing of putative regulatory sequences will be necessary to determine if *RBM24 *may play a role in this syndrome.

## Conclusion

Our data provide significant insight into the role of *rbm24 *in formation of cardiac and vascular systems, however much remains to be learned about the exact mechanism by which it functions and the role *RBM24 *may play in human disease. Other RRM containing proteins have also been shown to serve important roles in embryonic development [24,25] and post-transcriptional regulation is a frequent theme. With this in mind, we hypothesize that the protein plays a role in the post-transcriptional regulation of gene expression important for cardiac and vascular development. In the case of *rbm24a and rbm24b *depletion for instance, we found that expression of the ventricular marker *vmhc *is nearly abolished in the heart at 72 hpf, suggesting they both may contribute to specific regulation of *vmhc *mRNA. We will continue elucidate the role and mechanisms of *rbm24 *function in embryonic development. Furthermore, this study demonstrates the power of the screen that uncovered *Rbm24 *as a candidate cardiac gene, adding significant value to the original data set for which this work represents just the first of many such analyses awaiting completion.

## Methods

### Zebrafish maintenance

Adult AB zebrafish were maintained in system water according to standard methods [26]. Embryos were obtained from natural mating of adult fish. All experiments were in accordance with ethical permits by Johns Hopkins Animal Care and Use Committee under protocol number FI10M369.

### Bioinformatic identification of zebrafish rbm24 orthologs

We used the amino acid sequence of the protein encoded by the mouse *Rbm24 *gene to perform a blastp query searching the NCBI RefSeq database of *D. rerio *proteins from the Zv8 2008 genome assembly. Two genes were identified (*rbm24 *and *zgc:136803*) which encode proteins displaying strong similarity to the mouse Rbm24 protein (E-value = 5e-61; 103/116 (88%) and E-value = 3e-90; 188/237 (79%), respectively). As of the Zv9 2010 *D. rerio *genome assembly NCBI RefSeq now annotates the two genes we previously identified as two *rbm24 *paralogs with protein coding transcripts: *rbm24a *on chromosome 19 (RefSeq ID: NM_212865) and *rbm24b *on chromosome 16 (RefSeq ID: NM_001039925).

### Nucleic Acid in situ hybridization

Total RNA was isolated from whole zebrafish embryos at 24 hpf using **TRIzol **Reagent and total cDNA was generated with oligo-dT using the SuperScript III First-Strand Synthesis Kit (Invitrogen). Riboprobes for *rbm24a, rbm24b, myl7*, *myh6*, and *vmhc *were generated by PCR amplification of embryo cDNA. PCR fragments were TOPO cloned into PCRII vector (TA Cloning Kit Dual Promoter with pCRII vector, Invitrogen) and transformed into TOP 10 Cells (Invitrogen). Colonies were mini cultured and plasmid DNA was harvested using the QIAprep Spin Miniprep Kit (Qiagen) Digoxygenin-labeled riboprobes were synthesized from 1 μg plasmid DNA using Sp6 and T7 RNA Polymerase (DIG RNA Labeling Kit SP6/T7, Roche), and purified (SigmaSpin Colums, Sigma). Embryos for *in situ *hybridization at 48 hpf and older were treated with 0.003% 1-phenyl-2-thiourea (PTU) beginning at 48 hpf to reduce pigmentation. Embryos were fixed in 4% paraformaldehyde in PBS overnight at 4°C at 15.5 hpf, 24 hpf, 48 hpf, and 72 hpf; and *in situ *hybridization was performed as previously described [27].

### Morpholino design and injection

Antisense morpholinos for *rbm24a *and *rbm24b *were designed and provided by Gene Tools, LLC. The *rbm24a *translation blocking morpholino (5'-TGCATCCTCACGAAACGCTCAAGTG-3') and the *rbm24b *translation blocking morpholino (5'-AAATAAACTCCTTGCTCCTTGAAGG-3') were designed to hybridize to the 5' UTR immediately upstream of the translational start site. Morpholinos were diluted with dH_2_O to 20 ng/nL and titration experiments were conducted at 1, 2, 5 and 10 ng *rbm24a *MO and 1, 2.5, 5, 7 and 9 ng *rbm24b *MO to determine an effective dose (n = 100 embryos per concentration). Experimental concentrations of 5 ng *rbm24a *or 8 ng *rbm24b *MO were selected and injected into the yolks of 1-2 cell fertilized embryos (n = 75). Double knockdown experiments were conducted with co-injection of 2.5 ng *rbm24a *and 4 ng *rbm24b *translation blocking MO into embryos and compared to embryos injected with these amounts of either MO alone. The *rbm24a *splice blocking MO (5' CGTTATTTGAGATGCCTGACTGTT 3') and the *rbm24b *splice blocking MO (5' TATTTTGACGTTATTTACCTGGCTG 3') were designed to hybridize to the boundary of the second exon and second intron of the transcript and injected at 7.5 ng and 9 ng respectively. Antisense *p53 *MO (5'-GCGCCATTGCTTTGCAAGAATTG-3') previously published [28] was purchased from Gene Tools, LLC and 1 ng was injected into 1-2 cell stage fertilized embryos both with and without each *rbm24 *translation blocking MO (n = 50) [29]. Embryos were analyzed for cardiac and vascular phenotypes at 48, 72 and 96 hpf.

### RT-PCR to determine transcript knock down efficiency

Total RNA was isolated using **TRIzol **Reagent (Invitrogen) from whole morphant zebrafish embryos at 24 hpf after injection of either 7.5 ng *rbm24a *or 9 ng *rbm24b *splice blocking MO. Total cDNA was generated with oligo-dT using SuperScript III First-Strand Synthesis (Invitrogen). RT-PCR was carried out via the standard curve method on a Bio-Rad DNA Engine Opticon 2 Real-Time Detection System with primers specific to the correctly spliced transcripts at 100 ng input cDNA. Uninjected 24 hpf embryo cDNA was used to generate a standard curve and cDNA from uninjected experimental control embryos was assayed at 100 ng cDNA as the standard for 100% expression. All reactions were run in triplicate at 25 μL volumes using Power Sybr Green PCR Master Mix (1x final) (Applied Biosystems), primers at final concentration 0.08 μM each. Significance of expression reduction was determined using the students t-test statistic comparing transcript levels of *rbm24a *or *rbm24b *in uninjected controls to that measured cDNA from their respective morphants.

### *rbm24 *MO phenotype rescue

Total RNA was isolated from whole zebrafish embryos 24 hpf using **TRIzol **Reagent (Invitrogen) and total cDNA was generated with oligo-dT using SuperScript III First-Strand Synthesis (Invitrogen). Full length *rbm24a *and *rbm24b *cDNA were PCR amplified and cloned into the pCR8 gateway vector (pCR8⁄GW⁄TOPO TA Cloning Kit, Invitrogen) before being cloned into the pCSDEST destination vector (kindly provided by the lab of Nathan Lawson, UMass Med School). Full length sense capped Poly-A RNA was generated for *rbm24a *and *rbm24b *with the **mMESSAGE **mMACHINE kit (Ambion) and quantified with a NanoDrop 1000. Full length RNA was co-injected into 1-2 cell embryos with *rbm24a *MO or *rbm24b *MO at titrating levels and evaluated for phenotype rescue at 48, 72 and 96 hpf. Rescue was confirmed with 800 pg *rbm24a *RNA and 50 pg *rbm24b *RNA.

### Sequence analysis

Genomic DNA from two patients and an unaffected family member were included [23]. Direct sequencing of part of the promoter region (1031 bp), all exons plus exon-intron boundaries and a putative regulatory element in intron 3 (1164 bp) of the *RBM24 *gene was undertaken. Primers were designed to cover all four exons representing the "canonical" sequence (ENST00000379052, transcript length 2,458 bp and 236 aa, http://www.ensembl.org). PCR primers were designed by Primer3 software http://frodo.wi.mit.edu/cgi-bin/primer3/primer3.cgi and are available on request. Amplified PCR products were purified and sequenced using BigDye Terminator chemistry v3.1 on an ABI Prism 3130*xl *genetic analyzer (Applied Biosystems). Biomedical research involving human subjects has been performed according to the principles of the Helsinki's Declaration. Written informed consents were obtained from the patients and relatives involved in the research. Genomic DNA was taken from patients as per standard care and therefore ethical approval was not required.

### Heart Rate counts

Heart rates (beats per minute) were counted for uninjected, *rbm24a *MO and *rbm24b *MO injected embryos displaying the morphant phenotype at 48, 72 and 96 hpf. 50 embryos were analyzed per condition per time point. Average heart rates with standard error were plotted and significant heart rate deviation of morphants compared to uninjected controls was determined using the students t-test.

### Fluorescent vascular imaging

The transgenic zebrafish line TG(*kdrl*:G-RCFP) generated by Cross and colleagues [13] was used for vascular expression. Adult fish and embryos were maintained as described [26]. Embryos were injected with either 5 ng *rbm24a *MO or 8 ng *rbm24b *MO at the 1-2 cell stage and analyzed at 48 and 72 hpf for G-RCFP expression via fluorescence microscopy.

## Abbreviations

DA: dorsal aorta; PCV: posterior caudal vein; CV: caudal vein; hpf: hours post fertilization; RRM: RNA recognition motif; MO: morpholino antisense oligonucleotide; ISH: in situ hybridization; Se: intersegmetal vessels; DLAVs: dorsal longitudinal anastomotic vessels.

## Authors' contributions

SM and RM carried out *in situ *hybridization, morpholino analysis, experimental design and drafted the manuscript, while RM also designed antisense morpholinos. SM additionally conducted heart rate experiments, vascular experiments and assembled the figures and manuscript draft. SB carried out microinjections. DM contributed to *in situ *hybridization experimental data. MW, BD and AB conducted human RBM24 sequencing experiments on patient samples. ES, JG and SF provided intellectual input and experimental expertise. AM edited the manuscript, provided continuous experimental design, intellectual input and experimental expertise. All authors read and approve the final manuscript.

## Supplementary Material

Additional file 1 **Translation Blocking MO titrations**. Quantitative representation of percentage of embryos displaying cardiac phenotypes achieved from MO titrations. *rbm24a *MO 1, 2, 5 and 10 ng injected (A). *rbm24b *MO 1, 2.5, 5, 7 and 9 ng (B). Normal, looped beating heart with no cardiac edema; Class I, looped beating heart with cardiac edema; Class II, unlooped beating heart tube with cardiac edema; Class III, beating heart cell mass with cardiac edema; Dead, extreme cell death and degradation of embryo. n = 100 embryos per concentration. Click here for file

Additional file 2 **Uninjected zebrafish heart 48 hpf**. Digital video (time-lapse) captured of an uninjected control embryo (lateral) at 48 hpf. Movie demonstrates the looped structure and regular rhythm of the normal heart. Click here for file

Additional file 3 ***rbm24a *MO injected zebrafish heart 48 hpf**. Digital video (time-lapse) captured of an *rbm24a *MO injected embryo (lateral) at 48 hpf. Movie highlights the unlooped (linear) structure of the *rbm24a *morphant heart displaying edema at this stage in development. Atrial and ventricular chambers are still evident despite structural anomaly; however contraction is slow. Click here for file

Additional file 4 ***rbm24b *MO injected zebrafish heart 48 hpf**. Digital video (time-lapse) captured of an *rbm24b *MO injected embryo (lateral) at 48 hpf. Movie highlights the unlooped (linear) structure of the *rbm24b *morphant heart displaying edema at this stage in development. Atrial and ventricular chambers are still evident despite structural anomaly. Click here for file

Additional file 5 **Uninjected zebrafish heart 72 hpf**. Digital video (time-lapse) captured of an uninjected control embryo (lateral) at 72 hpf. Movie demonstrates the looped structure and regular rhythm of the normal heart, with circulation at this stage in development. Click here for file

Additional file 6 ***rbm24a *MO injected zebrafish heart 72 hpf**. Digital video (time-lapse) captured of an *rbm24a *MO injected embryo (lateral) at 72 hpf. Movie highlights the unlooped (linear) structure of the *rbm24a *morphant heart at this stage in development with cardiac edema. Heart atrial and ventricular chambers are difficult to distinguish and contracting with marked irregularity, with no circulation is detectable. Click here for file

Additional file 7 ***rbm24b *MO injected zebrafish heart 72 hpf**. Digital video (time-lapse) captured of an *rbm24b *MO injected embryo (lateral) at 72 hpf. Movie highlights the unlooped (linear) structure of the *rbm24b *morphant heart at this stage in development with additional cardiac edema. Atrial and ventricular chambers are still evident but highly distended, with no circulation is detectable. Click here for file

Additional file 8 **Uninjected zebrafish heart 96 hpf**. Digital video (time-lapse) captured of an uninjected control embryo (lateral) at 96 hpf. Movie demonstrates the looped structure and regular rhythm of the normal heart, with circulation at this stage in development. Click here for file

Additional file 9 ***rbm24a *MO injected zebrafish heart 96 hpf**. Digital video (time-lapse) captured of an *rbm24a *MO injected embryo (lateral) at 96 hpf. Movie highlights the unlooped (linear) structure of the *rbm24a *morphant heart at this stage in development with extreme cardiac edema. Heart atrial and ventricular chambers are difficult to distinguish and contracting with marked irregularity, with no circulation is detectable. Click here for file

Additional file 10 ***rbm24b *MO injected zebrafish heart 96 hpf**. Digital video (time-lapse) captured of severe example of one *rbm24b *MO injected embryo (lateral) at 96 hpf. Movie highlights the phenotype of the unlooped (linear) highly distended heart of *rbm24b *morphants at this stage in development, with no detectable circulation. The heart with distinct atrial and ventricular chambers is highly distended and contracting with marked irregularity, with no circulation is detectable. Click here for file

Additional file 11 ***rbm24a *and *rbm24b *splice blocked morphants display cardiac defects**. Injection of 7.5 ng of *rbm24a *splice blocking morpholino results in a substantial reduction of full-length transcript (A). Injection of 9 ng of *rbm24b *splice blocking morpholino results in aberrant splicing of the transcript. There is a reduction of full-length transcript 250 bp fragment and appearance of trace amounts of the shortened frameshift fragment 164 bp and shortened in-frame transcript 109 bp (B). RT-PCR measurement of transcript levels show both *rbm24a *(42.80% +/- 3.35, P < 6.5 × 10^-4^) and *rbm24b *(40.27 +/- 3.19, P < 4.4 × 10^-5^) morphants have significant reduction of transcript levels compared to uninjected controls (C) Error bars are standard deviation, *** *P *< 0.001. Click here for file

Additional file 12 **Supplemental Table 1**. Cardiac phenotypes displayed upon knockdown of *rbm24a *or *rbm24b *expression via splice blocking morpholino. Click here for file

Additional file 13 ***p53 MO *co-injection does not alter *rbm24a *and *rbm24 *morphant phenotypes**. Phenotypes were evaluated for embryos post injection *rbm24a *MO (5 ng) *or rbm24b *MO (8 ng) alone or in conjunction with *p53 *MO (1 ng) phenotypes were compared at 48, 72 & 96 hpf. Lateral heart views are shown with a dotted outline around embryo heart chambers. No cardiac phenotype is detected for *p53 *MO embryos compared to uninjected controls at any time point (A-B, G, H, M, N). At all time points *rbm24a *morphants maintain unlooped hearts and display cardiac edema in the presence of *p53 *MO (C, D, I, J, O, P). Morphant phenotype was also maintained between *rbm24b *morphants in the presence of *p53 *MO (E, F, K, L, Q, R). v, ventricle; a, atrium; black arrows, cardiac edema. Click here for file
