# Supplementary figures and images for: Identification of RNA binding motif proteins essential for cardiovascular development
Source: BMC Dev Biol. 2011 Oct 19;11:62. doi: 10.1186/1471-213X-11-62 (PMC3277282; doi:10.1186/1471-213X-11-62)

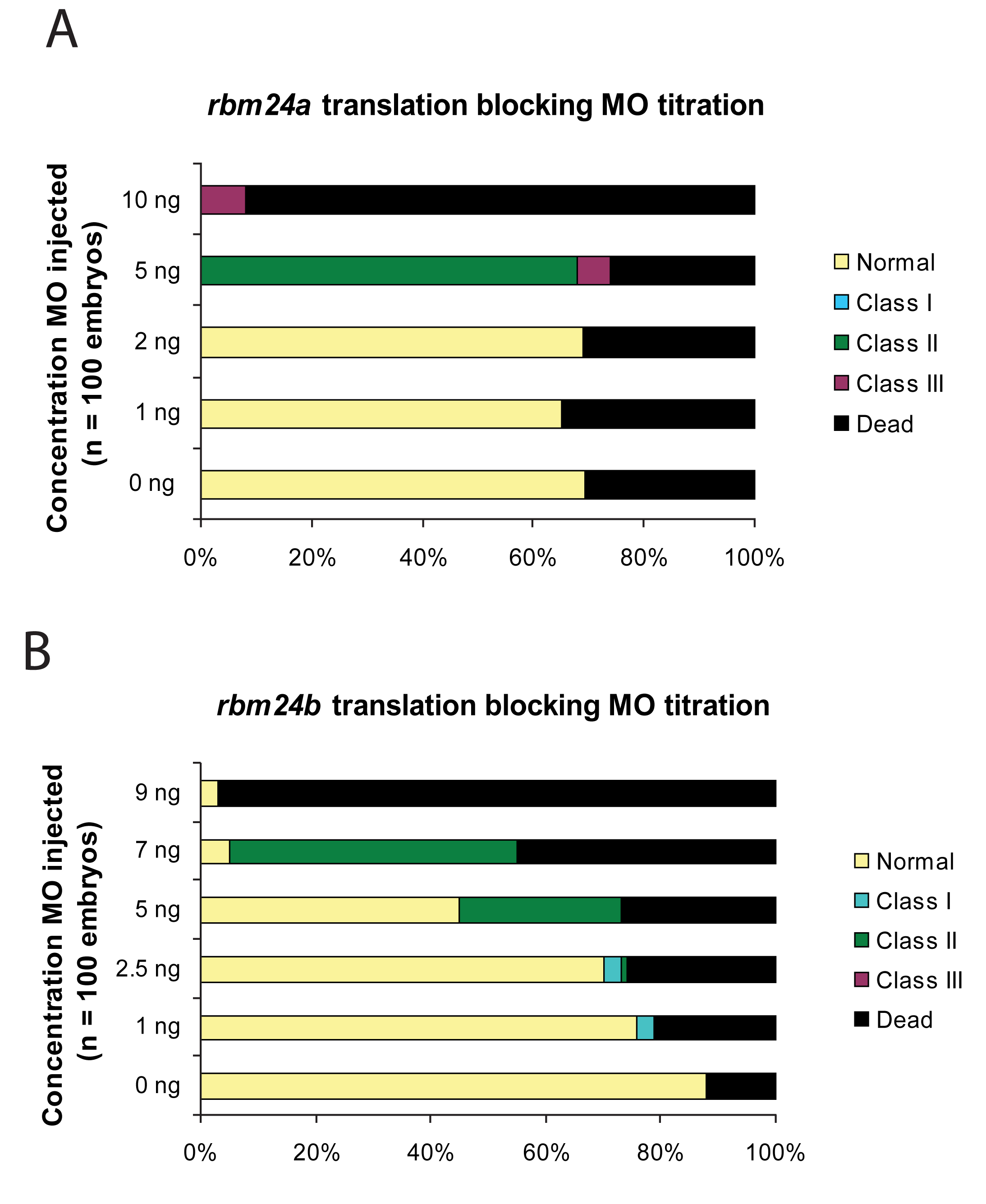

Supplement: Additional file 1 — Translation Blocking MO titrations. Quantitative representation of percentage of embryos displaying cardiac phenotypes achieved from MO titrations. rbm24a MO 1, 2, 5 and 10 ng injected (A). rbm24b MO 1, 2.5, 5, 7 and 9 ng (B). Normal, looped beating heart with no cardiac edema; Class I, looped beating heart with cardiac edema; Class II, unlooped beating heart tube with cardiac edema; Class III, beating heart cell mass with cardiac edema; Dead, extreme cell death and degradation of embryo. n = 100 embryos per concentration. [file 1471-213X-11-62-S1.jpeg]

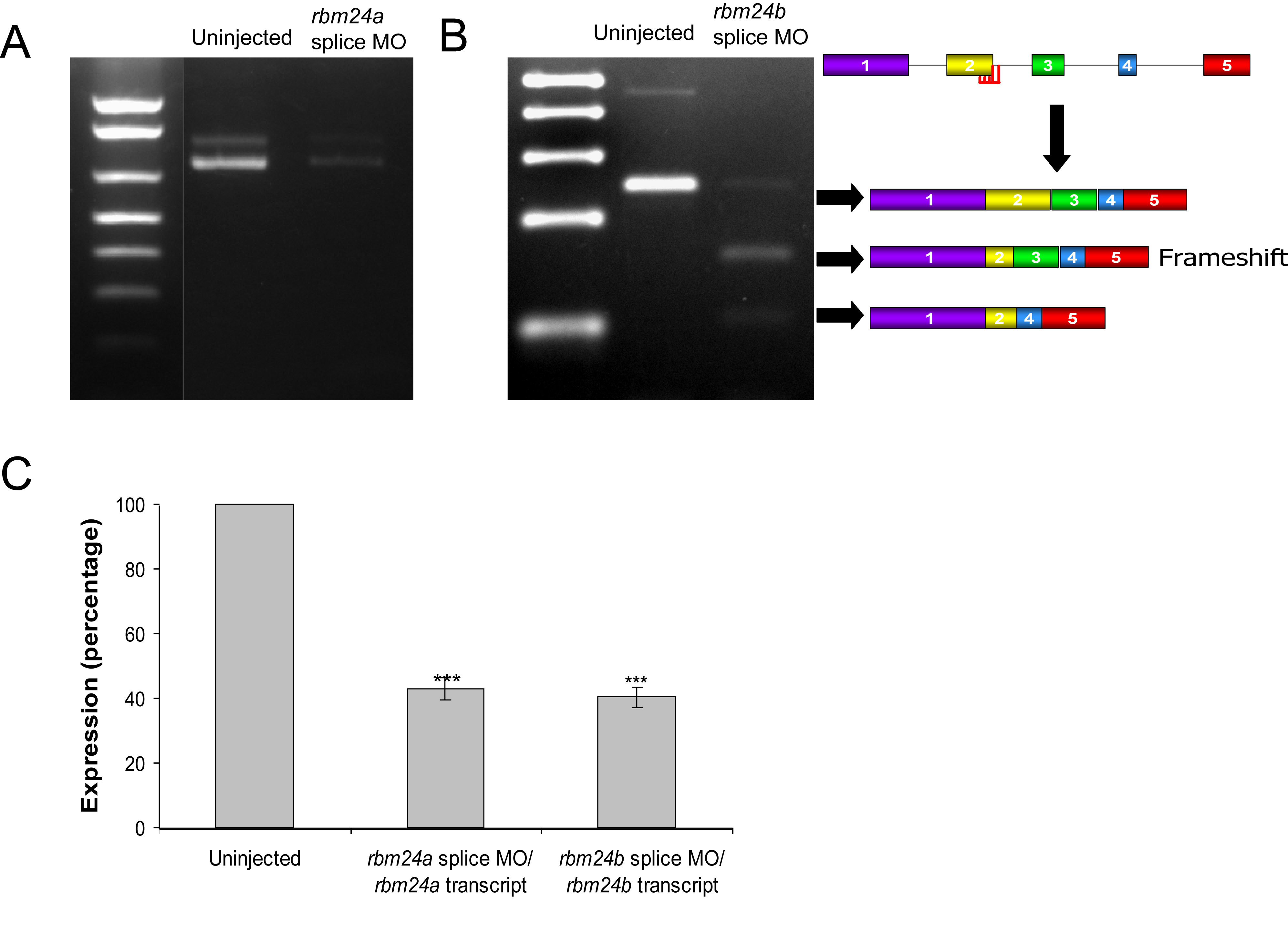

Supplement: Additional file 11 — rbm24a and rbm24b splice blocked morphants display cardiac defects. Injection of 7.5 ng of rbm24a splice blocking morpholino results in a substantial reduction of full-length transcript (A). Injection of 9 ng of rbm24b splice blocking morpholino results in aberrant splicing of the transcript. There is a reduction of full-length transcript 250 bp fragment and appearance of trace amounts of the shortened frameshift fragment 164 bp and shortened in-frame transcript 109 bp (B). RT-PCR measurement of transcript levels show both rbm24a (42.80% +/- 3.35, P < 6.5 × 10^-4^) and rbm24b (40.27 +/- 3.19, P < 4.4 × 10^-5^) morphants have significant reduction of transcript levels compared to uninjected controls (C) Error bars are standard deviation, *** P < 0.001. [file 1471-213X-11-62-S11.JPEG]

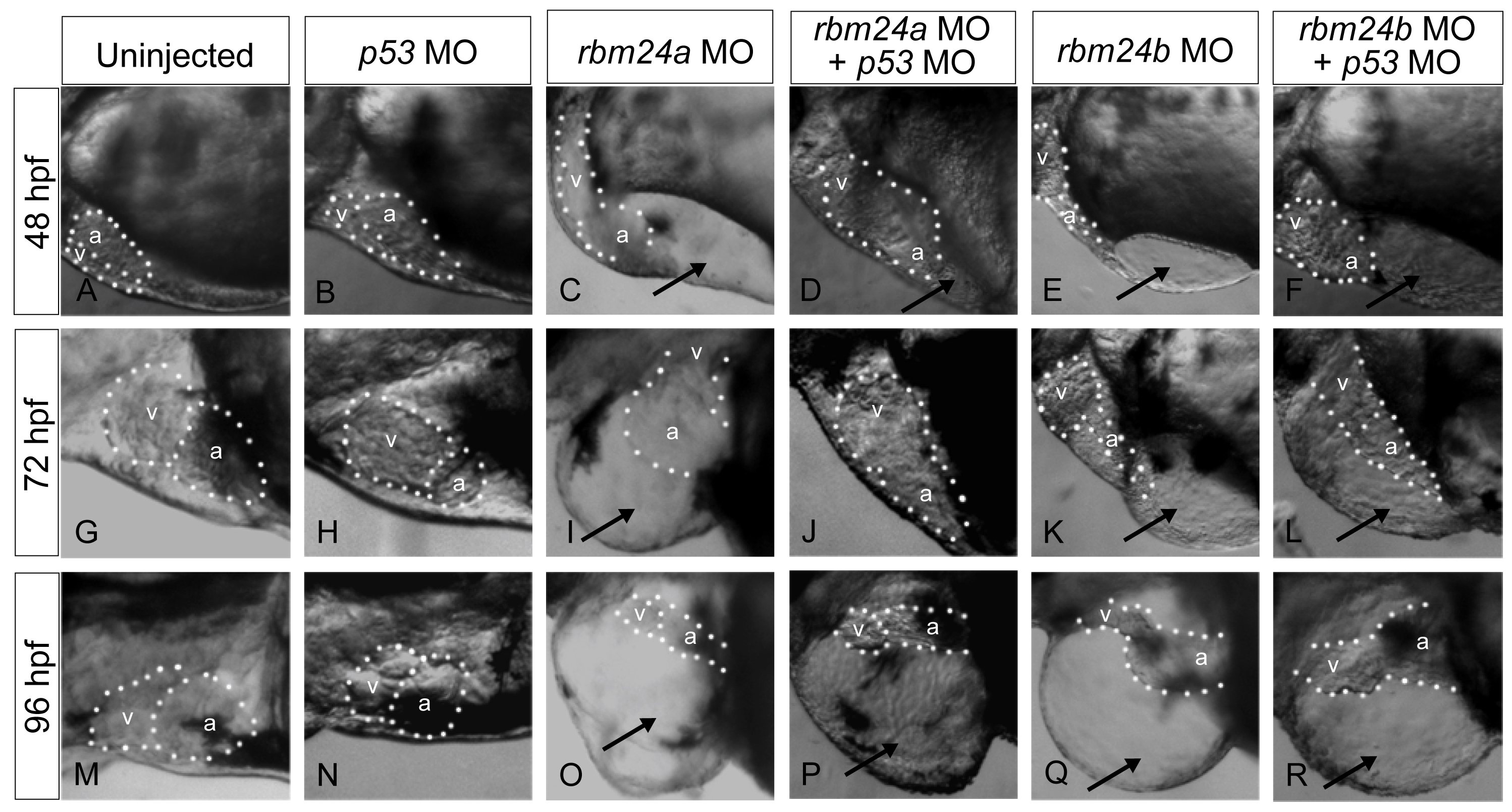

Supplement: Additional file 13 — p53 MO co-injection does not alter rbm24a and rbm24 morphant phenotypes. Phenotypes were evaluated for embryos post injection rbm24a MO (5 ng) or rbm24b MO (8 ng) alone or in conjunction with p53 MO (1 ng) phenotypes were compared at 48, 72 & 96 hpf. Lateral heart views are shown with a dotted outline around embryo heart chambers. No cardiac phenotype is detected for p53 MO embryos compared to uninjected controls at any time point (A-B, G, H, M, N). At all time points rbm24a morphants maintain unlooped hearts and display cardiac edema in the presence of p53 MO (C, D, I, J, O, P). Morphant phenotype was also maintained between rbm24b morphants in the presence of p53 MO (E, F, K, L, Q, R). v, ventricle; a, atrium; black arrows, cardiac edema. [file 1471-213X-11-62-S13.JPEG]
