# Supplementary material for: Identification of RNA binding motif proteins essential for cardiovascular development
Source: BMC Dev Biol. 2011 Oct 19;11:62. doi: 10.1186/1471-213X-11-62 (PMC3277282; doi:10.1186/1471-213X-11-62)
Supplement: Additional file 12 — Supplemental Table 1. Cardiac phenotypes displayed upon knockdown of rbm24a or rbm24b expression via splice blocking morpholino. [file 1471-213X-11-62-S12.PDF]

Table S1: Number of embryos analyzed for cardiac defects in *rbm24* splice blocking morphants

Morpholino	Dosage (ng)	Embryos Studied	Looping Defects	Cardiac Edema	No Cardiac Organization
rbm24a MOsb	7.5	96	89 (92.7 %)	89 (92.7 %)	0
rbm24b MOsb	9	79	43 (54.4 %)	43 (54.4 %)	0
